# INVESTIGATING THE USE OF FORMAL AND INFORMAL SUPPORTS BY OLDER ADULTS LIVING ALONE WITH MULTIPLE CHRONIC CONDITIONS

**DOI:** 10.1093/geroni/igad104.3444

**Published:** 2023-12-21

**Authors:** Morgan Minyo, David Bass, Katherine Judge, Catherine McCarthy

**Affiliations:** Cleveland State University, Cleveland, Ohio, United States; Benjamin Rose Institute on Aging, Cleveland, Ohio, United States; Cleveland State University, Cleveland, Ohio, United States; Benjamin Rose Institute on Aging, Cleveland, Ohio, United States

## Abstract

Older adults living alone with multiple chronic health conditions and/or functional limitations are at an increased risk for experiencing unmet needs and associated negative outcomes. Formal and informal support services benefit older adults by addressing unmet needs, making this population a common target among clinical interventions aiming to increase levels of support. To further our understanding of this vulnerable population, this investigation describes unmet needs and assistance with tasks in a sample of older adults living alone with chronic conditions (n=38). Baseline data collected from a randomized controlled pilot study was used for descriptive analyses. Participants average age was 77.1 (SD=11.6), 42.1% identify as Black, and 68.4% identify as female. On average, participants reported 6.0 (SD=3.7) chronic health conditions and 52.6% reported difficulty with two or more daily living tasks. Nearly 90% of participants reported at least one unmet need, with 60.5% having four or more (M=6.1, SD=5.2). Interestingly, 71.1% of participants reported receiving assistance from family/friends (M=2.8, SD=2.9) and 86.8% reported receiving assistance from service providers (M=3.1, SD=2.4) with at least one task in the past 3 months. Only one participant reported receiving no assistance. Family/friends most frequently assisted with household chores (47.4%), transportation (44.7%), and emotional support (39.5%). Service providers most frequently assisted with transportation (50.0%), emotional support (31.6%), and getting information about a health problem (31.6%). These findings provide insight on the formal and informal support services used by older adults who live alone and the individual characteristics that could make specific sub-groups successful clinical intervention targets.

